# Recent Advances in Divergent Synthetic Strategies for Indole-Based Natural Product Libraries

**DOI:** 10.3390/molecules27072171

**Published:** 2022-03-27

**Authors:** Taegwan Kim, Min Woo Ha, Jonghoon Kim

**Affiliations:** 1 Department of Chemistry, Soongsil University, 369 Sangdo-ro, Seoul 06978, Korea; lever19785@naver.com; 2 Integrative Institute of Basic Science, Soongsil University, 369 Sangdo-ro, Seoul 06978, Korea; 3 Jeju Research Institute of Pharmaceutical Sciences, College of Pharmacy, Jeju National University, 102 Jejudaehak-ro, Jeju 63243, Korea; 4 Interdisciplinary Graduate Program in Advanced Convergence Technology & Science, Jeju National University, 102 Jejudaehak-ro, Jeju 63243, Korea

**Keywords:** natural products, indole, small-molecule library, diversity-oriented synthesis, complexity-to-diversity (Ctd) strategy, biomimetic synthesis

## Abstract

Considering the potential bioactivities of natural product and natural product-like compounds with highly complex and diverse structures, the screening of collections and small-molecule libraries for high-throughput screening (HTS) and high-content screening (HCS) has emerged as a powerful tool in the development of novel therapeutic agents. Herein, we review the recent advances in divergent synthetic approaches such as complexity-to-diversity (Ctd) and biomimetic strategies for the generation of structurally complex and diverse indole-based natural product and natural product-like small-molecule libraries.

## 1. Introduction

The inherent biological activities of natural products are induced by specific and selective interactions with macromolecules in living organisms, which makes them potential bioactive compounds and drug candidates [1]. To allow facile access to natural product-based small-molecule modulators and potential therapeutic agents, natural product screening collections are essential [2]. Extensive investigations have been conducted for the isolation and synthesis of such screening collections [3,4,5,6]. However, the available collections of natural products are insufficient for processes such as high-throughput screening (HTS) and high-content screening (HCS), as they require hundreds of thousands of compounds [7].

To address the unmet needs associated with the construction of libraries which have a large number of natural products and related compounds, two different approaches, namely, combinatorial chemistry and diversity-oriented synthesis (DOS), have been broadly applied [8]. Natural product-based combinatorial chemical libraries are usually constructed by adding various building blocks to natural products with bioactivity and high synthetic accessibility in order to reduce the time and cost of drug candidate development [9]. Combinatorial chemistry has significantly contributed to the discovery and optimization of therapeutic compounds. However, the deficiency of skeletal diversity in natural product-based combinatorial chemical libraries could limit the application of undruggable targets and unbiased phenotypic screening [10,11]. Consequently, substantial synthetic efforts have been devoted to the construction of natural product-based libraries with a high degree of molecular skeletal and stereochemical diversity and complexity through the DOS strategy.

A synthetic approach based on the DOS strategy was introduced by Schreiber et al., allowing the construction of small-molecule libraries containing natural product-like scaffolds from simple starting materials through rapid synthetic transformations in a highly divergent and efficient fashion [12,13,14,15,16,17,18,19,20,21,22]. Various bioactive compounds were identified for undruggable targets and from unbiased phenotypic screening by screening DOS libraries of natural products and related structurally complex and diverse synthetic compounds, hence proving the robustness of this strategy.

Despite considerable success in the generation of natural product-based small-molecule libraries via the DOS strategy, there remains a demand for complementary synthetic approaches to various natural products and related compounds that provides greater structural diversity and complexity in a highly efficient manner. Thus, significant efforts towards the development of innovative synthetic approaches such as complexity-to-diversity (Ctd) and biomimetic synthetic strategies are underway. The purpose of this review is to briefly overview these two novel divergent synthetic strategies based on studies reported between 2014 and 2021. The discussion of DOS approaches for the generation of diverse natural-like compounds and discovery of bioactive compounds, found in the outstanding reviews of [14,18,20], are outside the scope of this review. In particular, indole is a biologically relevant structural heteroaromatic scaffold which is found in complex natural products and synthetic drugs [23,24,25,26,27,28]. The indole scaffold is a major target for the construction of small-molecule libraries for the discovery of therapeutic leads [29]. Several innovative synthetic methods and strategies have been developed to construct indole-based compound libraries to date. Thus, in this review we focus mainly on the divergent generation of indole-based natural products or related compounds via the Ctd and biomimetic synthetic strategies.

## 2. Complexity-to-Diversity Strategy for Rapid Generation of Indole-Based Natural Products and Related Compounds

**Figure 1:**
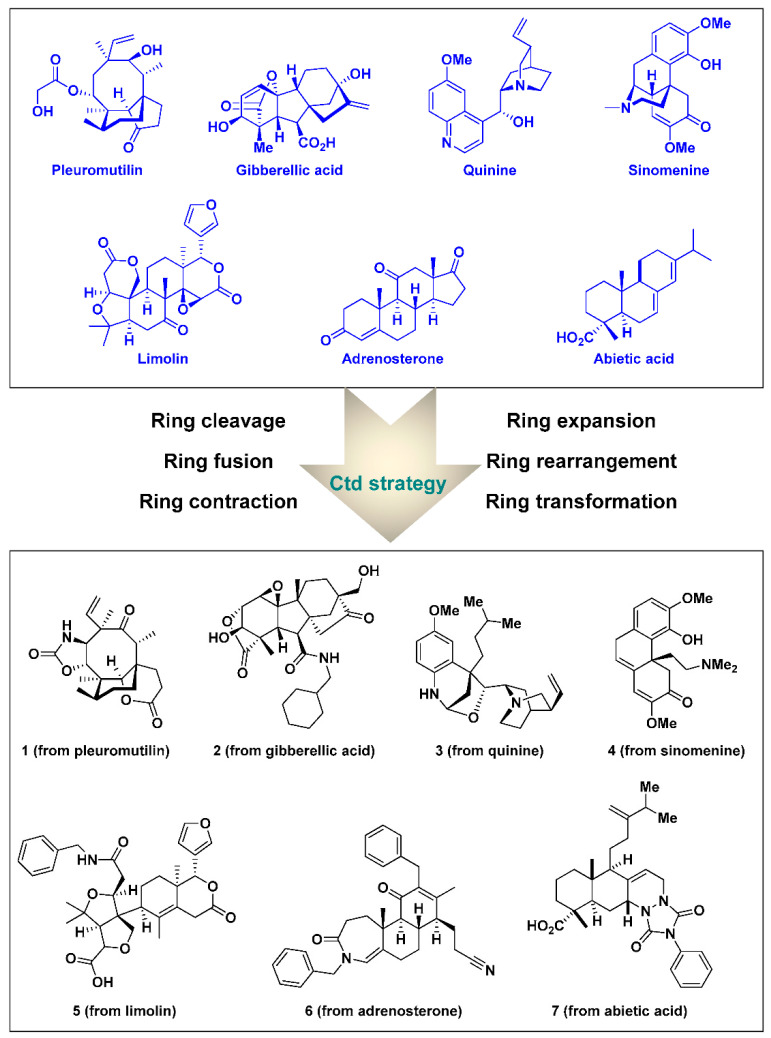
Natural products as starting points in the complexity-to-diversity (Ctd) strategy along with representative compounds generated via ring-distortion reactions.

In the typical total synthesis of natural products and the DOS approach for the generation of diverse natural products and related compounds, complex and intriguing structures of natural products are the goal and final target, not the starting point. However, the Ctd strategy considers the complexity and diversity in the synthetic scaffolds of the starting points. The Ctd strategy, introduced by Hergenrother et al. in 2013, employs systematic ring-distortion reactions for the construction of natural-product-like small-molecule collections using easily accessible complex natural products as starting points [30,31,32,33,34]. The Ctd approach achieves skeletal and stereochemical diversity via various chemoselective synthetic operations on the core ring region of the starting natural products while retaining their inherent stereochemistry and complexity. Biosynthetic pathways, with their delicate and efficient enzymatic routes which create complex and diverse natural products from common intermediates, inspired the development of the Ctd strategy. Moreover, owing to the structural complexity and uniqueness of the naturally-occurring skeletons in Ctd, diverse and discrete core structures can be easily generated through relatively simple chemical reactions such as ring expansion, contraction, breaking, and aromatization, as compared to the total synthesis of each natural product (Figure 1). Therefore, this strategy allows for the exploration of new biologically relevant chemical spaces. Initial reports have demonstrated structurally complex and commercially available natural products from diverse structural classes, such as abietic acid, adrenosterone, gibberellic acid, and quinine, as starting points for the Ctd strategy. The diversified core skeletons obtained via chemo-, regio-, and stereoselective chemical transformations have distinct 3D structural features which could have potential selective interactions with biopolymers. Indole alkaloids have been considered as a target and starting point for the Ctd strategy as well.

### 2.1. Ctd Strategy Based on Yohimbine (**8**)

Yohimbine (**8**) is a complex pentacyclic indole alkaloid extracted from the bark of the Pausinystalia tree [35]. It can act as an α2-adrenoceptor antagonist and is used to treat erectile dysfunction in men [36]. In 2017, Huigens et al. selected yohimbine (**8**) as the starting point for Ctd after considering its high complexity and easy availability [37].

**Scheme 1:**
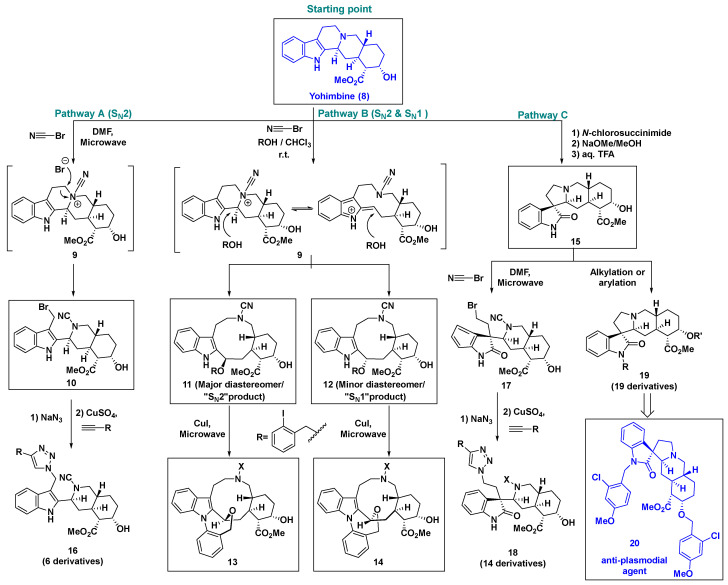
Ring distortion of yohimbine (**8**) for rapid access to complex and diverse indole-based natural product-like small molecules and identification of new anti-plasmodial agent compound **20**.

In order to systematically and efficiently alter the core ring of yohimbine (**8**), they devised a ring-distortion strategy based on two known synthetic transformations, namely, C-N bond cleavage [38] and oxidative rearrangement of tryptoline, a substructure of yohimbine [39] (Scheme 1). Chemoselective ring cleavage reactions are powerful tools for ring distortion. To this end, cyanogen bromide (CNBr) was used to activate the tertiary amine of yohimbine (**8**) to ammonium ion **9**, which could undergo orthogonal ring cleavage by different nucleophile attacks. Selective ring cleavage (pathway A, Scheme 1) occurred only in *N*,*N*-dimethylformamide (DMF) solvent, followed by bromide attack on the less hindered carbon via the S_N_2 pathway, resulting in the major product **10**. In contrast, the ammonium ion intermediary **9** formed the diastereomeric mixture of **11** and **12** by simultaneous S_N_1 and S_N_2 processes (pathway B, Scheme 1) in a chloroform/alcohol solvent mixture. Interestingly, after the introduction of 2-iodobenzyl alcohol, the diastereomers (**11** and **12**) underwent a Cu(I)-catalyzed C–N coupling reaction to afford the ring fusion products **13** and **14**, respectively. The ring rearrangement scaffold **15** was obtained via another tryptoline ring-distortion reaction involving oxidation and subsequent alkyl migration. Upon treatment with CNBr, spirooxindole **15** underwent selective C–N cleavage to afford compound **17**.

This Ctd strategy based on ring-distortion transformations of yohimbine (**8**) is effective for the rapid generation of new natural product-like scaffolds with high complexity. The subsequent typical diversification pathways, such as functional group introductions or click chemistry (often used in combinatorial chemistry), afforded as many as seventy products. The phenotype-based screening of this yohimbine-based library has successfully led to the identification of a new bioactive compound demonstrating antiproliferative activity against hypoxia-inducible factor (HIF)-dependent cancer cells [37]. In addition, compound **20** (Scheme 1) was recently found to be potent against chloroquine-resistant *Plasmodium falciparum* Dd2 cells (EC_50_ = 0.33 μM) [40].

### 2.2. Ctd Strategy Based on Vincamine *(**21**)*

**Scheme 2:**
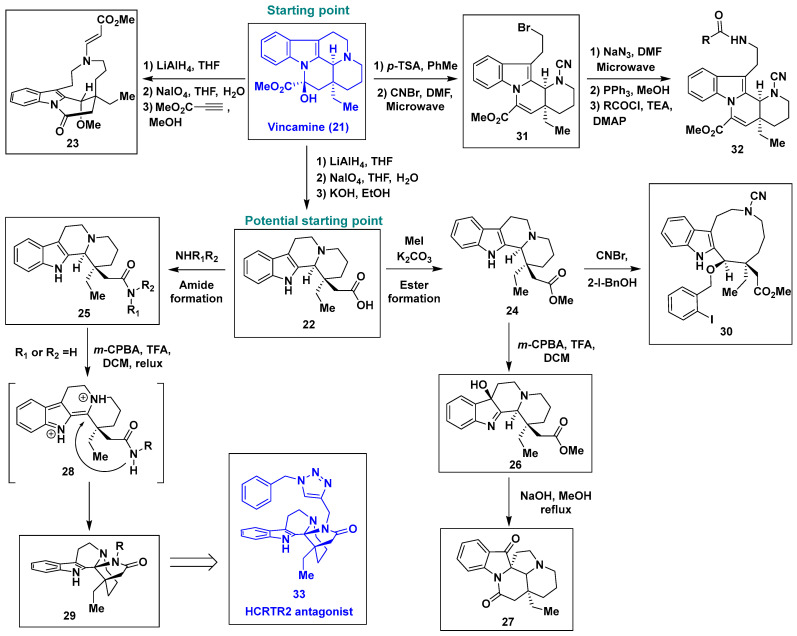
Ring distortion of vincamine (**21**) for rapid access to complex and diverse indole-based natural product-like small molecules and identification of a new HCRTR2 antagonist compound, **33**. *m*-CPBA, *meta*-chloroperbenzoic acid; 2-I-BnOH, 2-iodobenzyl alcohol.

Vincamine (**21**) is a commercially available monoterpenoid indole alkaloid administered to patients to enhance global or regional blood flow [41,42,43]. In 2020, Huigens et al. developed a remarkable Ctd strategy for vincamine (**21**), synthesizing eight stereochemically and structurally complex natural product-like scaffolds via the systematic application of a ring-distortion pathway including ring cleavage, ring rearrangement, and ring fusion reactions (Scheme 2) [44]. First, two reported ring cleavage reactions were employed to afford two new natural product-like compounds, **22** and **23**. Methyl propiolate was used to activate the tertiary amine of vincamine (**21**) to the ammonium ion, which was subsequently subjected to selective ring cleavage by methanol attack to form **23**. Notably, **22** became another useful starting point for the Ctd strategy. The carboxylic acid of **22** could be converted to either the ester, **24**, or the amide, **25**. Stereo- and regioselective oxidation of C3 in the indole core ring of **24** by *meta*-chloroperbenzoic acid (*m*-CPBA) afforded the hydroxylated compound **26**, which underwent base-promoted semipinacol rearrangement to yield the spirooxindole **27**. In contrast, the amide **25** underwent unexpected ring fusion upon treatment with *m*-CPBA to afford the novel compound **29**. It has been proposed that the selective oxidation of compound **25** to a 3-hydroxyindolenine intermediate followed by dehydration yields intermediate **28**, which undergoes 5-*exo*-trig cyclization to furnish **29**. In addition, CNBr-induced ring cleavage reactions yielded two new scaffolds, **30** and **31**, from compound **24** and vincamine (**21**), respectively. Subsequently, a typical diversified synthetic method was applied to the newly synthesized eight natural product-like compounds, which produced an eighty-member small-molecule library with distinct natural product-like complex scaffolds. The robustness of this Ctd strategy for vincamine (**21**) has been successfully validated by the identification of a new bioactive compound, **33**, which has antagonistic activity against hypocretin type 2 receptor (HCRTR2) and hence possesses therapeutic potential against opioid abuse [44].

### 2.3. Ctd Strategy Based on Nature-Inspired Indole Monoterpenoids

**Scheme 3:**
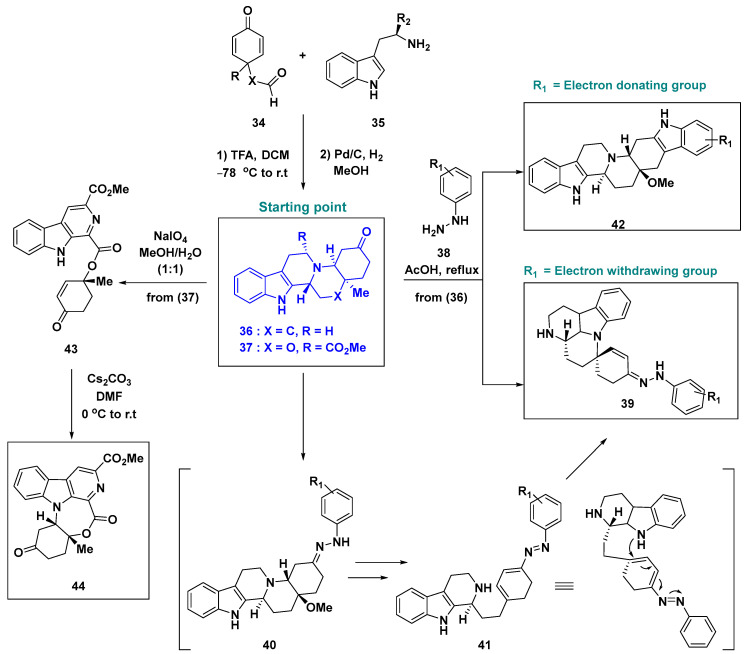
Ctd strategy based on nature-inspired indole monoterpenoids (**36** and **37**). Pd/C, palladium on carbon.

More recently, the Al-Tel group reported a new Ctd strategy for the nature-inspired indole monoterpenoid compounds **36** and **37** [45], prepared from compounds **34** and **35** [46] (Scheme 3). The compounds **36** and **37** have inherent complexity and transformable moieties such as cyclohexanone, piperidine, and indole rings, which makes them efficient starting points for the Ctd strategy. Using **36** and **37,** novel compounds such as indole [2,3-*a*]quinolizines (**42**), canthine-type alkaloids (**39**), and arborescidine-type alkaloids (**44**) were generated. Access to canthine-type scaffolds (**39**) was efficiently achieved by an unexpected reaction pathway upon treatment with electron-withdrawing-group-bearing hydrazines (**38**) in acetic acid. The proposed reaction mechanism involved the condensation reaction of compound **36** with phenylhydrazine (**38**) to yield the hydrazone intermediate **40**. Subsequent ring cleavage via the cascade reactions of tautomerization, ring-opening, and elimination of the methoxy group afforded the intermediate **41**. The diastereoselective intramolecular aza-Michael addition produced the canthine-type scaffold **39**. In contrast, the reaction of **36** with electron-donating-group-bearing hydrazines (**38**) proceeded via the regioselective [3,3] sigmatropic rearrangement of the hydrazone intermediates, thus generating octahydroindole[2,3-*a*]quinolizine scaffolds (**42**) via the typical Fischer–Indole synthetic pathway. When compound **37** was exposed to oxidation conditions, unexpected oxidative rearrangement occurred followed by the 7-*exo*-*trig* aza-Michael addition reaction, which afforded the new arborescidine-like indole alkaloid **44.**

## 3. Biomimetic Synthetic Divergent Approaches to Indole-Based Natural Product and Natural Product-Like Small Molecules

Biologically active natural products are among the best targets for the introduction of biological assay systems to explore unknown biological pathways, which may lead to potential therapeutic candidates [3,4,5,6]. However, synthetic accessibility is an obstacle owing to the stereo- and skeletal-complexity of natural products, despite notable advances in total synthesis and methodology. In contrast, natural product biosynthesis in living organisms allows facile access to structurally complex and distinct skeletons from relatively simple common intermediates through enzyme-catalyzed processes. This tremendous efficiency and diversity-generating power of the biosynthetic pathways allows for the rapid generation of diverse natural products and related compounds, including indole-based compounds [45,46,47,48,49,50,51,52].

**Scheme 4:**
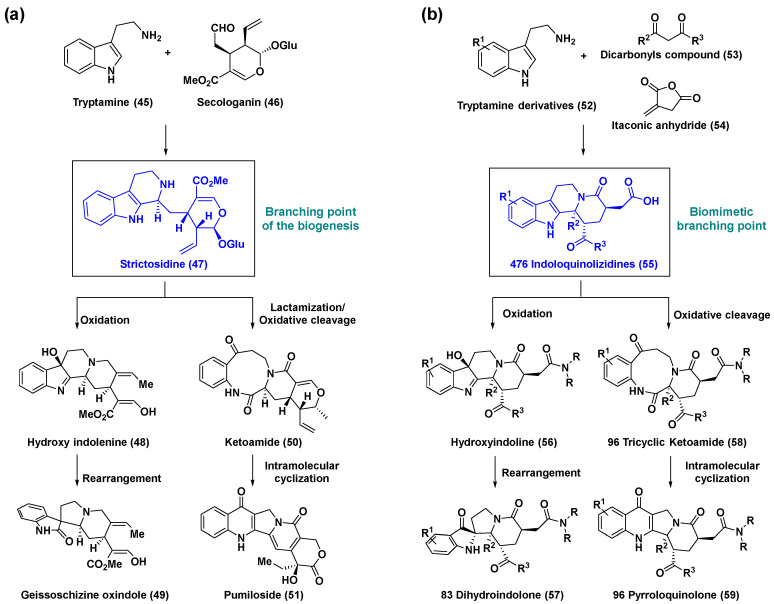
(**a**) Biosynthetic transformation of strictosidine (**47**), branching point of the biogenesis, and subsequent formation of monoterpenoid indole alkaloids. (**b**) Divergent synthetic pathways for the construction of an 847-member skeletally complex natural product-like compound library using a new biomimetic branching point, indoloquinolizidines (**55**).

A biosynthetic reaction of tryptamine (**45**) and secologanin (**46**) yields the key intermediate and a biosynthetic branching point strictosidine (**47**), a precursor to other structurally diverse and biologically significant monoterpene indole alkaloids formed via the biosynthetic pathways of structural rearrangement, oxidation, and intramolecular cyclization [53]. Thus, strictosidine (**47**) makes an attractive biomimetic branching point to furnish structurally distinct and complex skeletons. Consequently, substantial research efforts were aimed at developing new biomimetic branching points. The Kozmin group developed a new pluripotent synthetic branching point, indoloquinolizidine (**55**), which was readily assembled from tryptamine derivatives (**52**), 1,3-dicarbonyl compounds (**53**), and itaconic anhydride (**54**) (Scheme 4) [54]. Key intermediates (**55**) were designed based on strictosidine (**47**), which could undergo a highly controlled diversification process to afford complex skeletal natural products including hydroxy indolenine (**48**), spirocyclic geissoschizine oxindole (**49**), ketoamide (**50**), and pumiloside (**51**) (Scheme 4a). The biosynthetic pathways of strictosidine (**47**) were mimicked by transforming the indoloquinolizidines (**55**) to skeletally diverse scaffolds such as hydroxyindoline (**56**), dihydroindolone (**57**), tricyclic ketoamide (**58**), and pyrroloquinolone (**59**) (Scheme 4b). This bioinspired synthetic strategy successfully generated an 847-member small-molecule library. More such biomimetic pathways have been reported in the literature [29].

### 3.1. Development of a New Biomimetic Divergent Branching Point from Dehydrosecodine *(**61**)*

**Scheme 5:**
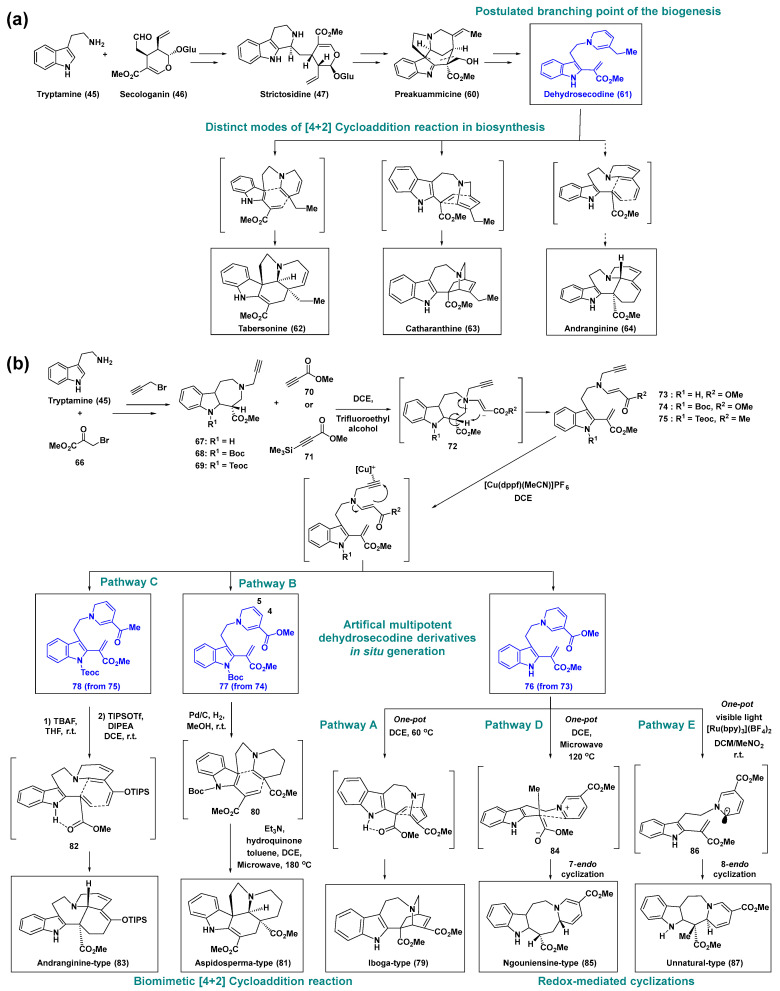
(**a**) Biosynthetic transformation of preakuammicine (**60**) via the postulated branching point, i.e., intermediate **61** to monoterpenoid indole alkaloids. (**b**) Biomimetic route for the preparation of monoterpenoid indole-type alkaloids via the transformation of dehydrosecodine derivatives (**76**–**78**), which act as biomimetic branching points. dppf, 1,1′-bis(diphenylphosphino)ferrocene; bpy, 2,2′-bipyridine; Teoc, 2-(trimethylsilyl)-ethoxycarbonyl.

Preakuammicine (**60**), synthesized from strictosidine (**47**), may be rearranged by two C–C bond cleavages to provide one of the most important intermediates in biosynthesis, dehydrosecodine (**61**), which comprises a dihydropyridine and a vinylindole. This intermediate, **61**, allows easy access to generate the aspidosperma-type alkaloid tabersonine (**62**), iboga-type catharanthine (**63**), and andranginine (**64**), respectively, through distinct modes of [4 + 2] cycloaddition reactions (Scheme 5a) [55,56]. Despite its utility, dehydrosecodine (**61**) has not been isolated or synthesized because of its high reactivity and instability under oxidation conditions, which restrict its practical applications. Therefore, dehydrosecodine derivatives are an attractive option to design desired biomimetic pathways.

Oguri et al. developed new artificial pluripotent dehydrosecodine derivatives (**73**–**75**), which were subjected to biomimetic [4 + 2] cycloadditions and redox-mediated cyclizations to generate five architecturally complex scaffolds (Scheme 5b) [57]. The introduction of additional electron-withdrawing substituents such as carbonyl groups allows feasible access to dehydrosecodine derivatives (**73**–**75**) and controls their regio- and stereoselectivity in the systematic intramolecular synthetic progress. First, the reactions of tryptamine (**45**) with other building blocks yielded tricyclic compounds (**67**–**69**), which were then converted to en-yne compounds (**73**–**75**) upon treatment with methyl propiolate (**70**) or 4-(trimethylsilyl)-3-butyn-2-one (**71**), respectively, via the formation of zwitterionic intermediate (**72**) and subsequent regioselective Hofmann elimination. The en-yne compounds (**73**–**75**) underwent 6-*endo* cyclization to generate dihydropyridine-vinylindole intermediates (**76**–**78**). This reaction pathway mimicked that of dehydrosecodine (**61**), without further intra- and intermolecular reactions of the resultant π-conjugated system in the presence of a Cu(I) catalyst. Interestingly, the en-yne compound **73** was transformed to the iboga-type compound **79** upon treatment with a Cu(I) catalyst in 1,2-Dichloroethene (DCE) at 60 °C via a one-pot cascade dihydropyridine formation and subsequent [4 + 2] cycloaddition reaction (Pathway A).

In contrast, compound **74**, possessing a Boc group at the indole N1 position, was transformed to the dehydrosecodine derivative **77** by Cu(I)-catalyzed dihydropyridine formation without a subsequent cycloaddition. It was suggested that the hydrogen at the indole N1 position forms a hydrogen bond with the carbonyl oxygen of the α,β-unsaturated methylester, resulting in the increased electrophilicity of the dienophile and fixed orientation, thus rendering the subsequent [4 + 2] cycloaddition reaction more pronounced. Based on this observation, compound **74** was subjected to Cu(I)-catalyzed dihydropyridine formation; the subsequent site-selective hydrogenation of the C4-C5 double bond furnished an isolable tetrahydropyridine intermediate, **80**, which did not lead to in situ iboga-skeleton formation or dihydropyridine oxidation (Pathway B). Finally, in the presence of hydroquinone and trimethylamine, the tetrahydropyridine intermediate **80** underwent a distinct mode of [4 + 2] cycloaddition followed by thermal decomposition of the Boc group (180 °C in a microwave) to furnish the aspidosperma-type skeleton compound **81**.

Furthermore, the tricyclic compound **69**, with a 2-(trimethylsilyl)-ethoxycarbonyl (Teoc) group on the indole nitrogen, was converted upon treatment with 4-(trimethylsilyl)-3-butyn-2-one (**71**) into en-yne compound **75**, possessing a methyl ketone group. The resultant en-yne compound, **75**, underwent Cu(I)-catalyzed dihydropyridine formation to afford the dehydrosecodine derivative intermediate **78**, which was then transformed to silyl enol ether intermediate, **82**, when the Teoc group was removed. The distinct mode of [4 + 2] cycloaddition spontaneously produced the andranginine-type scaffold **83** (Pathway C).

Notably, the dihydropyridine-vinylindole intermediate **76** was found to be capable of undergoing redox-mediated cyclization reactions as well as [4 + 2] cycloaddition. The ngouniensine-type alkaloid **85** was produced by microwave-assisted heating of the dihydropyridine-vinylindole intermediate **76** at 120 °C. Based on the experimental results of deuterium-labeled dihydropyridine-vinylindole intermediate **76** and subsequent structural confirmation, a mechanism was proposed to the effect that the hydride of the dihydropyridine-vinylindole intermediate **76** shifts to form the zwitterionic intermediate **84**, which possesses a pyridinium cation and an enolate anion, and subsequently undergoes 7-*endo* cyclization to furnish the ngouniensine-type alkaloid **85**. Furthermore, upon treatment with the photo-redox catalyst [Ru(bpy)_3_](BF_4_)_2_ under visible-light irradiation at 0 °C, the dihydropyridine ring of **76** underwent single-electron oxidation and was converted to the radical species **86**, which generated an unnatural tetracyclic scaffold, **87**, via redox-mediated 8-*endo* cyclization (Pathway E). This study successfully validated the utility of dehydrosecodine derivatives acting as biomimetic branching points to generate natural product-like small-molecule libraries without structural simplification.

### 3.2. Development of New Biomimetic Divergent Branching Points to Form Monoterpene Indole Alkaloids

**Scheme 6:**
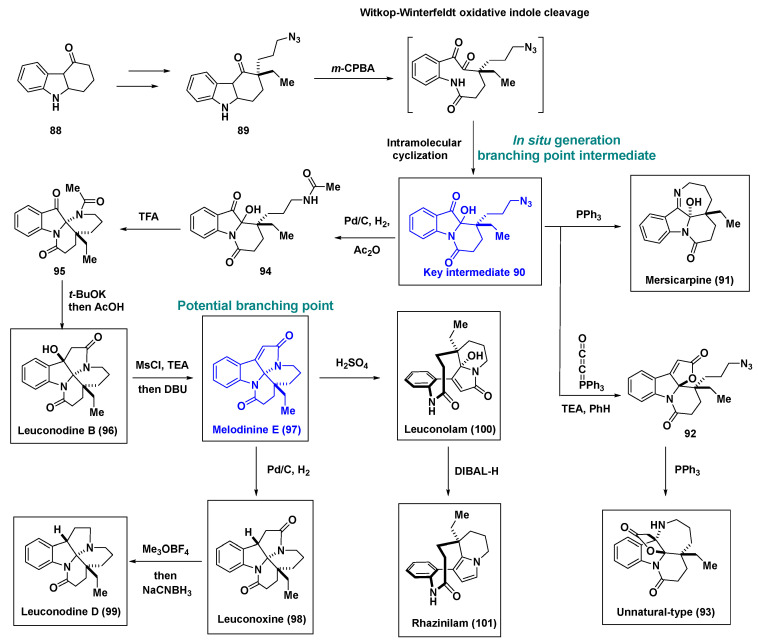
Divergent synthetic pathways to generate seven natural product and natural product-like monoterpene indole alkaloids using a new biomimetic branching point, intermediate **90**. DBU, 1,8-diazabicyclo 5.4.0 undec-7-ene; DIBAL-H, diisobutylaluminium hydride; MsCl, methanesulfonyl chloride.

Dai et al. developed a new pluripotent intermediate, **90**, for a divergent synthetic approach to natural or unnatural monoterpene indole alkaloids [58] (Scheme 6). Compound **89** engaged in a cascade involving Witkop–Winterfeldt oxidative indole cleavage and transannular cyclization upon treatment with *m*-CPBA, leading to the key intermediate **90**, which served as a synthetic branching point. Intermediate **90** was unstable in nature and was immediately subjected to the divergent synthetic pathway without purification or with rapid purification. For example, in situ-generated intermediate **90** could be subjected to the Staudinger-aza-Wittig process in the presence of triphenylphosphane (PPh_3_), yielding mersicarpine (**91**). Furthermore, following the Bestmann ketene lactonization of in situ-generated intermediate **90**, the resultant lactam **92** was, upon treatment with PPh_3_, transformed into the unprecedented indole-based skeleton compound **93**.

To generate different monoterpene indole alkaloids, melodinine E (**97**) was used as another pluripotent branching point. To synthesize melodinine E (**97**), the azide of intermediate **90** was first converted to the acetamide of compound **94**. Upon treatment with TFA, cyclization occurred to afford compound **95**, which was then converted into leuconodine B (**96**) via an intramolecular aldol reaction. Leuconodine B (**96**) underwent dehydration to afford melodinine E (**97**), which yielded leuconoxine (**98**) upon catalytic hydrogenation. Chemoselective reduction of the five-membered lactam upon treatment with Meerwein’s salt (Me_3_OBF_4_) and sodium cyanoborohydride furnished leuconodine D (**99**). In contrast, the addition of H_2_SO_4_ induced the rearrangement of melodinine E (**97**) to leuconolam (**100**), which on chemoselective DIBAL-H reduction of the 5-hydroxy-pyrrolone yielded rhazinilam (**101**). Notably, this study provided new insights into the potential biosynthetic pathways for terpene indole alkaloids and the feasibility of constructing indole-based natural products and natural product-like small-molecule libraries via the development of artificial pluripotent intermediates.

### 3.3. Biomimetic Divergent Synthesis to Generate Iboga and “Post-Iboga” Alkaloids

**Scheme 7:**
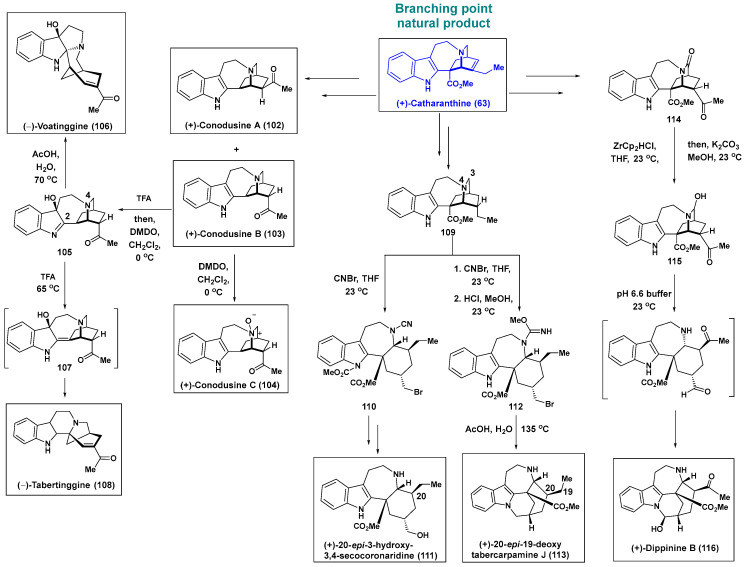
Biomimetic routes for the preparation of various iboga and “post-iboga” alkaloids from (+)-catharanthine (**63**) as the biomimetic branching point. ZrCp_2_HCl, bis(cyclopentadienyl)zirconium chloride hydride; DMDO, dimethyldioxirane.

Iboga alkaloids are a large family of monoterpenoid indole alkaloids that contain indole, tetrahydroazepine, and isoquinuclidine scaffolds. Following the 1901 isolation of ibogaine, an iboga alkaloid possessing anti-addictive activity, considerable progress has been made in the identification of new ibogaine-type natural products over the past 100 years, leading to the identification of hundreds of iboga alkaloids and the development of remarkable synthetic pathways for them [59,60,61,62]. In addition, secondary metabolites produced from iboga alkaloids via biosynthetic pathways have sparked great interest. In 2019, inspired by the efficient biosynthesis of secondary metabolites with iboga alkaloids as the starting point, Han et al. developed a biomimetic method for the transformation of (+)-catharanthine (**63**) to various iboga alkaloids and secondary metabolites coined “post-iboga” alkaloids (Scheme 7) [63]. In that study, (+)-catharanthine (**63**), a starting material for the synthesis of the anticancer drug navelbine, was used as a biomimetic branching point with high accessibility.

First, (+)-catharanthine (**63**) was converted to (−)-conodusine A (**102**) and (+)-conodusine B (**103**). Dimethyldioxirane (DMDO) conveniently and selectively oxidized the tertiary amine group of (+)-conodusine B (**103**) to produce (+)-conodusine C (**104**). In contrast, when the tertiary amine group of **103** was protected by TFA, as well as upon subsequent addition of DMDO, hydroxyindolenine **105** was obtained. Interestingly, it was demonstrated that hydroxyindolenine, **105**, is converted into “post-iboga” alkaloids such as (−)-voatinggine (**106**) and (−)-tabertinggine (**108**) through distinct structural reorganizations under acidic conditions. Hydroxyindolenine **105** was transformed into (−)-voatinggine (**106**) upon treatment with acetic acid. The proposed reaction mechanism entails acid-mediated β-elimination of N4 in hydroxyindolenine **105**, followed by nucleophilic attack on C2. In contrast, when hydroxyindolenine **105** was subjected to TFA at 65 °C, (−)-tabertinggine was generated. The proposed mechanism involves the tautomerization of hydroxyindolenine **105** to the enamine derivative **107**. The subsequent protonation of the tertiary alcohol group and azafulvenium ion formation and reorganization yielded(−)-tabertinggine (**108**).

To advance different “post-iboga” alkaloids, CNBr was introduced to cleave the C3–N4 bond. Compound **109**, synthesized from (+)-catharanthine (**63**), underwent selective C3–N4 bond cleavage upon treatment with CNBr, producing the ring cleavage product **110**. Compound **110** was transformed into the C20-epimer (+)-3-hydroxy-3,4-secocoronaridine (**111**), one of the “post-iboga” alkaloids. CNBr-induced ring cleavage of **109** yielded the carbamimidate compound **112**, which underwent hydrolysis intramolecular alkylation to furnish pentacyclic chippiine- and dippinine-type “post-iboga” alkaloids such as 20-epi-19-deoxytabercarpamine J (**113**). Finally, selective reduction of the lactam moiety in **114** (generated from (+)-catharanthine (**63**) through a synthetic sequence) using a Schwartz reagent led to the formation of the unstable hemiaminal compound **115**, which underwent rearrangement to form (+)-dippinine B (**116**) in a pH 6.6 buffer solution. The skeletally diverse and complex secondary metabolites of iboga alkaloids and “post-iboga” alkaloids obtained from the starting point of (+)-catharanthine (**63**) confirms the robustness of this strategy. In addition, the study provided new insights into the potential biosynthetic pathways for “post-iboga” alkaloids.

## 4. Conclusions

Natural products with highly complex and diverse structures are desirable candidates for screening bioactivity owing to their remarkable specificity toward biomolecular targets. However, the current natural product screening libraries are insufficient for processes such as HTS and HCS. To address this issue, a DOS strategy has been used to generate natural products and drug-like compounds with high skeletal and stereochemical diversity. Despite significant advances in DOS strategies, efficient synthetic methods for the rapid and easy preparation of small-molecule libraries comprising complex and diverse natural products remain challenging. Herein, we briefly reviewed Ctd and biomimetic synthetic strategies for the efficient generation of indole-based natural product and related small-molecule libraries. These two strategies enable high molecular diversity and structural complexity in the small-molecule libraries, thus distinguishing themselves as powerful synthetic tools. However, high demand for more starting points and branching points will always remain. The synthesis and identification of new pluripotent synthetic starting points could be a promising way to advance new complex natural products and related small-molecule libraries.

## Data Availability

Not applicable.
